# FluDetWeb: an interactive web-based system for the early detection of the onset of influenza epidemics

**DOI:** 10.1186/1472-6947-9-36

**Published:** 2009-07-29

**Authors:** David Conesa, Antonio López-Quílez, Miguel Ángel Martínez-Beneito, María Teresa Miralles, Francisco Verdejo

**Affiliations:** 1 Departament d'Estadística i Investigació Operativa, Universitat de València, 46100 Burjassot (Valencia), Spain; 2 Centro Superior de Investigación en Salud Pública, 46020 Valencia, Spain; 3 Área de Epidemiología, Conselleria de Sanitat, Generalitat Valenciana, 46020 Valencia, Spain; 4 Consultoría Promedio, 46006 Valencia, Spain

## Abstract

**Background:**

The early identification of influenza outbreaks has became a priority in public health practice. A large variety of statistical algorithms for the automated monitoring of influenza surveillance have been proposed, but most of them require not only a lot of computational effort but also operation of sometimes not-so-friendly software.

**Results:**

In this paper, we introduce FluDetWeb, an implementation of a prospective influenza surveillance methodology based on a client-server architecture with a thin (web-based) client application design. Users can introduce and edit their own data consisting of a series of weekly influenza incidence rates. The system returns the probability of being in an epidemic phase (via e-mail if desired). When the probability is greater than 0.5, it also returns the probability of an increase in the incidence rate during the following week. The system also provides two complementary graphs. This system has been implemented using statistical free-software (ℝ and WinBUGS), a web server environment for Java code (*Tomcat*) and a software module created by us (*Rdp*) responsible for managing internal tasks; the software package *MySQL *has been used to construct the database management system. The implementation is available on-line from: http://www.geeitema.org/meviepi/fludetweb/.

**Conclusion:**

The ease of use of FluDetWeb and its on-line availability can make it a valuable tool for public health practitioners who want to obtain information about the probability that their system is in an epidemic phase. Moreover, the architecture described can also be useful for developers of systems based on computationally intensive methods.

## Background

Public Health agencies use disease surveillance tools in order to monitor the incidence or prevalence of specific health problems over time. This knowledge allows them to detect changes in the estimated incidence rates, which produces better planning and allocation of resources and the possibility of avoiding breakdowns in Health Care Systems. In addition, a good surveillance infrastructure can be very useful in preparing for pandemics and for monitoring new emerging diseases.

An important matter of concern when dealing with the surveillance of infectious diseases is that of detecting the onset of an epidemic as soon as possible. The early identification of infectious disease outbreaks would enable prompt intervention which could have, for example, a great impact on the number of lives saved. Several statistical methods have been proposed (and most of them applied) over recent decades for detecting outbreaks and informing health authorities of the presence and spread of disease (see LeStrat [1], Buckeridge [2] and Burkom [3] for comprehensive surveys of these kinds of methods and Bravata et al. [4] for a critical evaluation of the potential utility of surveillance systems for illnesses and syndromes related to bioterrorism up to that date).

Among other diseases, influenza has been of special interest among researchers as influenza epidemics occur virtually every year and result in substantial disease, death and expense. Moreover, genetic changes in the influenza virus make vaccine effectiveness questionable every year and give this disease pandemic potential. Although the extent and severity of such epidemics vary greatly, it is worth noting that approximately 10–15% of people get influenza around the world every year and that the disease is responsible for up to 50 million illnesses and up to 47,200 deaths in the United States each year, with a similar situation in Europe , [5][6][7]. With all these figures in mind, it is quite understandable why the control of influenza has become a priority in public health practice.

As a result, a large variety of statistical algorithms for the automated monitoring of influenza surveillance have been proposed. The most widely used approaches are based on historical limit methods or on Serfling's method [8]. For instance, these methods are used, respectively, in Europe by the European Influenza Surveillance Scheme (EISS) and in the United States by the Center for Disease Control and Prevention (CDC) Influenza Branch. Although both methods are very easy to implement, they have some drawbacks (see Rath et al. [9] and Martínez-Beneito et al. [10] for more details). Many other solutions have been proposed and we just highlight here some of the most recent: LeStrat and Carrat [11], Rath et al. [9], Viboud et al. [12], Cowling et al. [13], Nuño and Pagano [14], Bock et al. [15] and Jégat et al. [16].

The complexity of disease surveillance methods has been increasing progressively. In fact, most of the above mentioned methods are not easy to implement. On the contrary, most of them and, in general, most advanced surveillance systems require skilled personnel to implement, fine-tune and maintain them. These requirements have kept these new developments from common usage. In order to resolve this issue, there has been a recent interest in enhancing existing disease surveillance methodologies by using tools for presenting data and information to users. Hauenstein et al. [17] describe in detail the processes and tools (such as system architecture, web-based applications, etc.) needed to do so. Two examples of how web-based surveillance systems can enhance the ability for identifying, estimating and assessing public health hazards are a web application by Pelat et al. [18], which allows users to analyze seasonal time series with periodic regression models, and Berchialla et al. [19], who present a web-based tool for injury risk assessment of foreign body injuries in children. Lewis et al. [20] review other existing automated disease surveillance systems in use by health departments (ESSENCE, RODS, EARS, RedBat and SYRIS).

The main purpose of this paper is to provide an enhanced web implementation of a novel prospective influenza surveillance methodology [10]. The method uses a Bayesian Markov switching model to determine the epidemic and non-epidemic periods from influenza surveillance data, and so detect influenza epidemics during the first onset week or as soon as the data allow. Nevertheless, this methodology requires a lot of computational effort and knowledge of sometimes not-so-friendly software. In particular, in order to estimate the parameters of the model, Markov Chain Monte Carlo (MCMC) methods are necessary, WinBUGS [21] being our choice to carry out the inference.

Implementation of the surveillance methodology has been done using a client-server architecture with a web-based client application design. By way of a friendly interface, users can introduce and edit their own data consisting of a series of weekly influenza incidence rates. Users may also obtain estimates of the probability of being in an epidemic phase for weeks of interest. The estimation process is not immediate, so the system has been designed to respond to requests from a multi-user environment on a first-come, first-served basis. After completion of the process, the system returns the probability of being in an epidemic phase together with the probability of an increase in the incidence rate during the following week. It also provides two graphs. The first one shows the weekly rates of the last two seasons indicating whether the posterior probability of being in an epidemic phase in the analyzed week is greater than 0.5 or not. The second one shows all the weekly rates with flags only for requested weeks. In particular, flags indicate whether the posterior probability of being in an epidemic phase is greater than 0.5 or not. The ease of use and its on-line availability should make the resulting application a valuable tool for public health practitioners.

## Implementation

In what follows, we introduce the kind of data sets that could be analyzed using our prospective surveillance method [10], we briefly review the method itself and we describe the client-server architecture and the client application design used to implement our surveillance methodology.

### Data

The method was originally developed to analyze data from the Valencian Sentinel Network (VSN) for influenza surveillance, a system which collects information on influenza-like illness (ILI) in the Comunitat Valenciana, one of the 17 autonomous regions in Spain. Like other sentinel Networks, the VSN is formed by volunteer practitioners that report weekly the number of ILI cases (usually defined as fever plus acute respiratory symptoms such as cough and/or sore throat) in seasons (each one lasting 30 weeks) that extend over two consecutive years, as the epidemic activity usually extends across both of them. It is worth mentioning that each weekly rate is obtained by considering the population covered by those sentinels that report information on the corresponding week.

**Figure 1:**
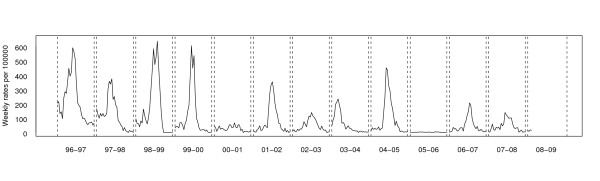
**Valencian Sentinel Network data**. Time series of the weekly influenza incidence rates (per 100.000 inhabitants) during the eleven seasons (from 1996–1997 to 2008–2009) analyzed.

The resulting data consist of various time series formed by the weekly ILI incidence rates (per 100.000 inhabitants) provided by the VSN during the seasons of interest. As an example, Figure 1 displays thirteen time series formed by the weekly ILI incidence rates provided by the VSN during the seasons from 1996–1997 to 2008–2009 (this latter not being complete). Note that the behaviour of the incidence rates cannot be strictly considered as seasonal because of the low rates observed in the fifth and tenth seasons. The main reason for the low rates is that there was no virus circulating (as confirmed by the absence of virus isolates during those weeks). Clearly, some bias is introduced here: the volunteer practitioner who notifies the ILI cases could act differently in front of similar situations. But, this variability is constant with time, and so we think that it does not incorporate any problem in order to detect if the system is in an epidemic phase.

Nevertheless, the usefulness of a surveillance method is measured by its adaptability to the environment in which it operates. As stated above, our method was developed to analyze weekly incidence rates (as is usual in all the Spanish Sentinel Networks). But it can be adapted (with slight modifications) to work with data coming from Sentinel Networks in which providers report weekly the percentage of patients with ILI from the total number of patients seen and the number of those patients with ILI. Moreover, the method is applicable not only for Western countries, but for any other network in which the identified periods of high possibility of influenza activity last the whole year. In this latter case, seasons could be defined as the whole year.

### Underlying methodology

Instead of modelling the mean of the influenza incidence rates series, it has been discussed in [10] that it is more appropriate to model the first-order differenced series (formed by the differences between rates in consecutive weeks). In particular, the underlying prospective influenza surveillance method is based on a modelling which segments the series of differences into two phases, epidemic and non-epidemic, using a Markov switching model (see [10] for a detailed description of the method).

In particular, if *Y *= {*Y*_*i*, *j*_, *i *= 1,..., nweeks - 1; *j *= 1,..., nseasons} denote the set of differences between the rates of consecutive weeks, each *Y*_*i*, *j *_is associated with an unobserved random variable *Z*_*i*, *j *_that indicates which phase the system is in (1, epidemic; 0, non-epidemic), the unobserved sequence of differences following a two-state Markov chain of order 1 with transition probabilities:

Inference on *Z *(the epidemic vs. non-epidemic state) for every week is the main goal of our application. The key point is that the conditional distribution of the differences (except for the first one) is modelled either as an autoregressive process of order 1 with high variability or as a Gaussian white noise process of lesser variability depending on whether the system is in an epidemic or non-epidemic phase:

Using all the data set, Bayesian paradigm is used to estimate the parameters, which needs the specification of the priors and their corresponding hyperpriors (see [10] for more details). Nevertheless, the resulting posterior distribution of the parameters *P *(parameters|data) does not yield analytical estimates and so in order to estimate the parameters of the model, Markov Chain Monte Carlo (MCMC) methods are necessary, WinBUGS [21] being our choice to carry out the inference. More details and the WinBUGS code can be downloaded from the following web page: http://www.geeitema.org/doc/meviepi/influenza.html. From the simulation of the posterior distribution of all the parameters it is possible to obtain a lot of information. In particular, it can be used to identify which are the epidemic weeks during the whole period analyzed, most importantly, the distribution of the state of the last week analyzed. Knowing whether the system is in an epidemic phase during the analyzed week is so important because it allows an on-line use of the method which can be crucial to detecting the time step at which the epidemic phase starts.

Still more information can be obtained from the simulation of the posterior distribution. In particular, when the system is in the epidemic phase, it could be interesting to predict if in the following week there would be an increase in the rate (indicating that the analyzed week is previous to the peak in the analyzed season) or whether there would be a decrease in the rate (indicating that the peak has already been reached). Within the Bayesian paradigm, prediction of an unknown observable (in this case, the next difference of rates) is done via its posterior predictive distribution (posterior because it is conditional on the observed and predictive because it is a prediction for an observable), that is:

where *P*(*Y*_*i*, *j*_|parameters) ~ .

Neither the posterior predictive distribution nor the posterior distribution of the parameters have an analytical form. Nevertheless, it is not difficult to obtain a simulation from the predictive distribution *P*(*Y*_*i*, *j*_|data) by first simulating from the posterior distribution of the parameters *P*(parameters|data) and then simulating from the distribution of the difference *Y*_*i*, *j *_conditional to those previously simulated valuesof the parameters (see, for instance, Gelman et al. [22] for a description of how to simulate from posterior predictive distributions).

### Architecture of the system

As Hauenstein et al. [17] state, "the cornerstone of a robust and effective electronic information system is a carefully designed architecture that meets the needs of its users for reliability, performance, and usability and the requirements of the development team for cost, scalability, security and maintainability".

Following their comments, one of the first issues to consider when building an information system is to choose an appropriate architecture.

**Figure 2:**
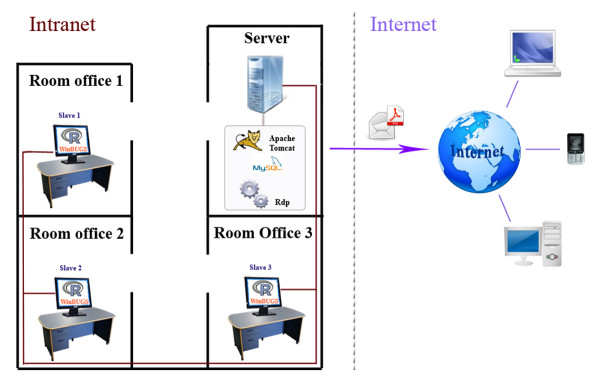
**Architecture of the system**. Description of the intranet connections between computers (each one based in a different room office) of the department where the system is based, jointly with the internet connection of the server with the rest of potential users via the world wide web.

In our case, although our algorithm can be installed in a simple stand-alone system in which any user can deal with his/her own data, data-specific adjustment can be tricky. To facilitate general usage, we have implemented our software so that multiple users may share the application simultaneously by communicating with a server over a network connection. This architecture is usually known as client-server. We have implemented a thin client application design for ease of user interaction with our program, that is through a web application that could be accessed by any network-enabled device (PC's, PDA's or cell phones) with a web browser. But moreover, the computational requirements of our detection algorithm, which could need several minutes to return the results (as it requires a MCMC simulation process) has made us use a master-slave intranet architecture in order to take advantage of the available computers (with usual statistical software – ℝ [23] and WinBUGS [21] – installed) in our department. In particular, as can be appreciated in Figure 2, there are various computers acting as slaves and connected via intranet with the server, which acts as the master.

**Figure 3:**
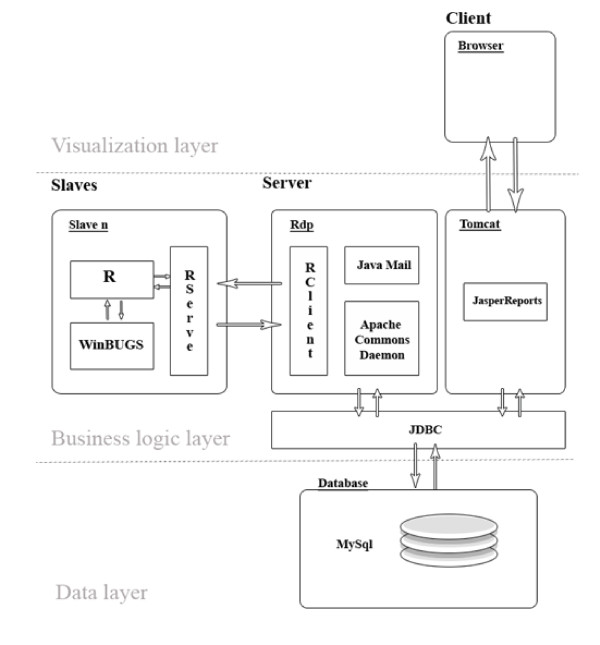
**Internal architecture of the system**. Implementation of the system has been made as a three-tier architecture by separating its functions into three separate layers: the presentation layer (responsible for interaction between users and the system), the business logic tier (in charge of the running of the algorithm) and the data tier (responsible for data storage).

Figure 3 contains information about the internal architecture of our server and its connections with the slaves and clients. The system has been implemented as a three-tier architecture by separating its functions into three separate layers. The top tier corresponds to the presentation layer and is responsible for interaction between the user and the system through data and personal information querying, visualization of results, etc. This has been done via a website with dynamic content programmed using JavaServer Pages (JSP) [24], a Java technology that allows software developers to dynamically generate HTML, XML or other types of documents in response to a Web client request.

The second tier is the business logic tier, which is the core of the system as it controls the running of our prospective influenza surveillance algorithm. This tier consists of two components. The first one is *Tomcat *[25], a web container that functions as a web application server supporting servlets and JSPs whose function is to insert and edit data in the database and send information to the visualization tier. The second one is *Rdp *(R Distributed and Persistent), a software module created by us and implemented in Java using *Apache Commons Daemon*, *Rclient *and *Java Mail *libraries. We call it "distributed" because tasks are distributed between slaves, and "persistent" because all the necessary information for recovering the system is stored in the database via the Application Programming Interface (API) *JDBC*.

Basically, *Rdp *is responsible for managing tasks and controlling the availability of slaves in order to send tasks to those free slaves and recover information from them when the task is finished. In particular, when a request for the probability of being in the epidemic phase is sent by any user, the request is stored in the database in a list of tasks to be done. *Rdp *is in charge of checking both the list of tasks and the list of free slaves in such a way that when *Rdp *detects that there is one free slave and one task on the list, it sends the task to the slave to be done. As the process to complete the tasks is not immediate, the system has been designed to respond to demands on a first-come first-served basis. The *Rclient *module is used to connect the server with *R-serve*, a package of ℝ installed in each slave. This package is ultimately responsible for sending the tasks to ℝ and WinBUGS. When the task is done, the results obtained are sent (if desired) to the user attaching a pdf document generated using the API *JasperReports *[26].

Using all the computers in the department to make the calculations allows any member of the departament to check the list of tasks to be done at any moment and (if necessary) execute *Rserve *on his/her PC and add the PC to the list of free slaves.

The final layer is the data tier and, as mentioned above, it is responsible for data storage, not only of the influenza rates but also of the user's personal information, availability and state of slaves, IP addresses, assigned tasks, etc. In order to construct our relational database, we have used MySQL^© ^software [27].

## Results

In what follows we present a case study to demonstrate how our web-based application allows users (epidemiologists, public health officials, etc.) to obtain the posterior probability of being in an epidemic phase, and so rapidly detect when the annual flu epidemic period starts. To do that, we will use the data set introduced above, consisting of the thirteen time series formed by the weekly ILI incidence rates provided by the VSN during the seasons from 1996–1997 to 2008–2009. All the WinBUGS and ℝ codes are freely available in Additional file 1.

### Using the system

After registering (when using the system for first time) and logging on, users automatically enter the initial page from which they can access the four main pages. From the first page, users can edit and modify their personal information, while the second page is from where users can enter and/or edit their own influenza data. As mentioned above, weekly ILI incidence rates must be per 100.000 inhabitants.

**Figure 4:**
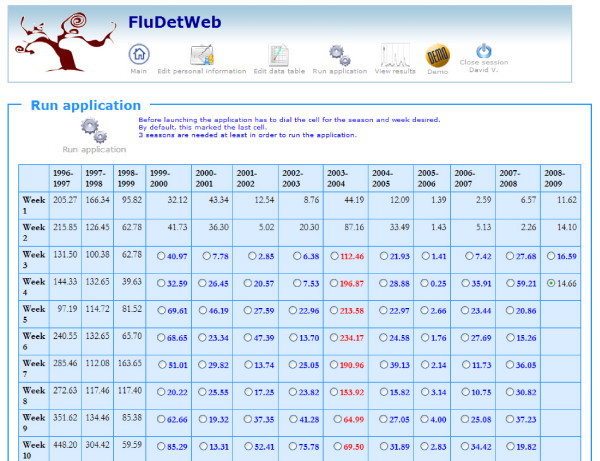
**Application launcher page**. Table with all the data (weekly incidence rates) from where users can request for the probability of being in the epidemic phase. Red and blue weekly incidence rates indicate epidemic and non epidemic weeks, respectively. Black weekly incidence rates are those in which it is not possible to perform the algorithm. Black bold stands for a week in which it is possible to launch it.

The third page give access to the application launcher. This page consists of a table with all the data (weekly incidence rates) from where users can request for the probability of being in the epidemic phase for the last week introduced. It is worth noting that in order to apply the whole mechanism of the Bayesian paradigm discussed in the previous section, the number of analyzed series must be greater than three. The reason is that the method needs to have enough data in order to learn about the disease's behaviour. Although the main usefulness of FluDetWeb is to determine whether the epidemic phase has begun in the analyzed week, it can also be valuable for users to get this information for any previous week. This capability allows computation of week-to-week sensitivity and specificity of the algorithm if laboratory test or other confirmation is available. In other words, we can use FluDetWeb to obtain the posterior probability of being in the epidemic phase at any other moment in the past only taking into account information from the weeks previous to that instant. In this case, the system keeps track of all the resulting probabilities and indicates in the application launcher page in which weeks it is not possible to obtain the posterior probability (because there is not enough data to do so), in which ones it has not been obtained and, for those in which it has been calculated, if probability is greater than 0.5 (showing the weekly rate in red) or not (in blue). Note that this use of the system corresponds to the one that users will follow if they keep incorporating new data each week and obtaining the probability of being in the epidemic phase with the new data. Figure 4 shows how this page would look when dealing with the VSN data set.

The process for obtaining the results could take several minutes, depending on how busy the system is. If users select the option "Send results via e-mail" in the personal data, they will get the results in a pdf file. A second option is to look at the View Results page when calculations are finished. This page is similar to the application launcher page, but instead of showing the rates it shows the posterior probability of being in the epidemic phase (with the same code of colors mentioned above) for all the weeks in which we have asked to obtain it (following the above mentioned condition of using only information from the weeks previous to the one analyzed). FluDetWeb shows a separate page of results for each week analyzed. This page presents the posterior probability of being in the epidemic phase. Values exceeding 0.5 indicate that, in that week, we are observing a higher probability of being in an epidemic phase than of being in a non-epidemic one, and so an alarm could be triggered if considered necessary. If this probability does exceed 0.5, the program also shows the probabilities of an increase and of a decrease in the incidence rate for the coming week. Otherwise, no other probabilities are shown.

This information should be sufficient for users to detect when the annual flu epidemic period starts. But bearing in mind that the best way of communicating information to users is by using visualization components [17], FluDetWeb also provides two graphs. The first one is a comparison graph of the weekly influenza incidence rates during the current and the previous season indicating if the posterior probability of being in an epidemic phase in the analyzed week is lower than 0.5 (black spot) or greater (red spot). The second one shows the weekly rates of all the seasons and indicates, in a similar manner to the application launcher page, in which weeks it is not possible to obtain the posterior probability (showing the weekly rate in black), in which ones it has not been obtained (in white), and, for those in which it has been calculated, if probability is greater than 0.5 (in red) or less than 0.5 (in blue).

### Analyzing the data from the VSN

The Valencian Sentinel Network collects weekly ILI incidence rates in seasons that extend over two consecutive years, each season lasting 30 weeks (from the 42nd week of one year to the 19th week of the following), and has been reporting information on ILI cases since 1996. As can be appreciated in Figure 1, at the time of writing this paper (October 29th, 2008), data consist of twelve complete time series (from 1996–1997 to 2007–2008) and one partial time series (corresponding to the 2008–2009 season) only containing four weekly ILI incidence rates.

**Figure 5:**
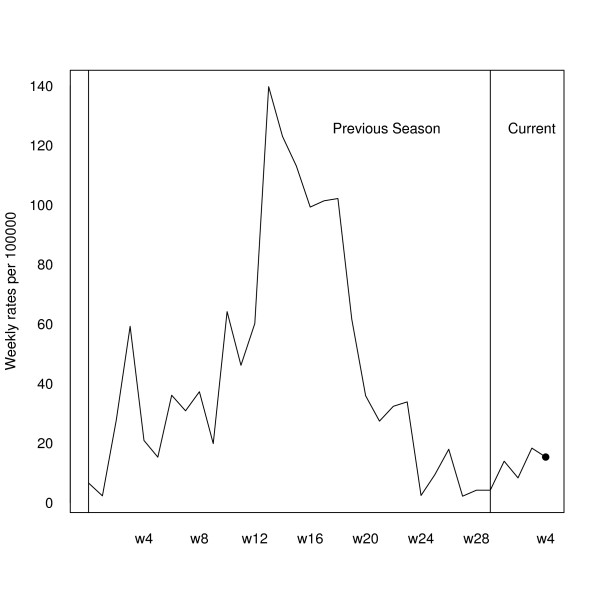
**Analysis of the fourth week of the 2008–2009 season**. Weekly influenza incidence rates (per 100.000 inhabitants) during the current season and the previous one indicating that in this week (the fourth of the current 2008–2009 season) the posterior probability of being in an epidemic phase is lower than 0.5.

**Figure 6:**
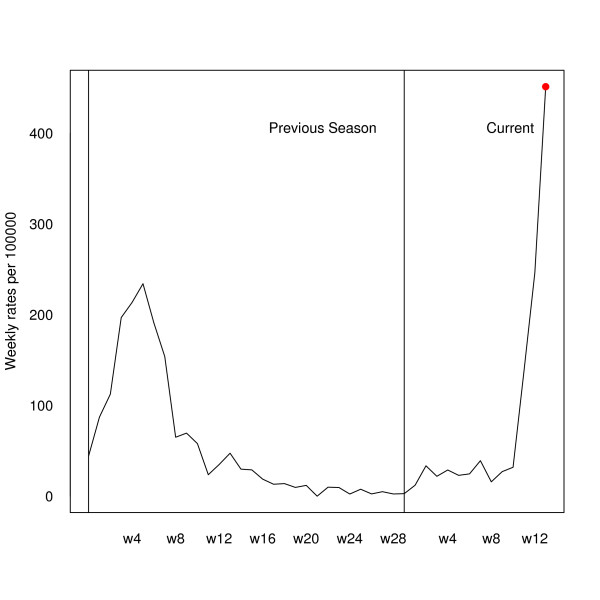
**Analysis of the 13th week of the 2004–2005 season**. Weekly influenza incidence rates (per 100.000 inhabitants) during the 2004–2005 season and its previous one indicating that in that week (the 13th of the 2004–2005 season) the posterior probability of being in an epidemic phase was greater than 0.5.

Let us suppose that we are a first time user of FluDetWeb. When registering we should indicate that the number of weeks per season is 30. After introducing the data set, our main interest would be to know if the epidemic phase has begun. After launching the application, the system returns that the posterior probability of being in the epidemic phase in the fourth week of the 2008–2009 season is 0.012, thus indicating that the epidemic phase has not begun. As this probability does not exceed 0.5, the system does not show the probabilities of an increase and of a decrease in the incidence rate for the coming week. This information is completed with a graph that shows the weekly influenza incidence rates during the current and the previous season, indicating that in the analyzed week (the fourth one of the current 2008–2009 season) the posterior probability of being in an epidemic phase is lower than 0.5. Figure 5 shows this graph. Usage of the system in subsequent weeks will be the same. Every week we would have to add the new weekly incidence rate and with the new data we could have control over the behaviour of the annual influenza epidemic. But, in order to show how FluDetWeb behaves, we have also calculated the posterior probability of being in the epidemic phase in other previous instants (taking into account information only up to that moment). In particular, as an example, we have obtained the posterior probability of being in the epidemic phase for the 13th week of the 2004–2005 season. The value obtained is 1, showing that at that moment the system was in an epidemic phase. As this probability is greater than 0.5, we have also calculated the posterior predictive distribution of the following difference between rates, from where we can assess that the conditional probability of an increase in the following week was 0.75 for that week (0.25 being the probability of a decrease). In other words, at that moment the epidemic was still growing. Figure 6 shows the comparison of weekly rates of seasons 2003–2004 and 2004–2005, and the weekly rate of the 13th week of the 2004–2005 season in red, thus indicating a posterior probability of being in the epidemic phase greater than 0.5.

**Figure 7:**
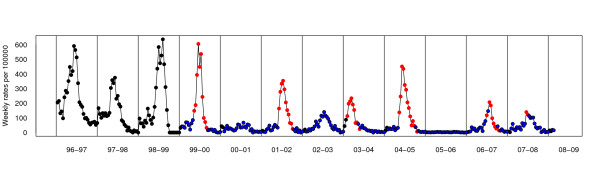
**Analysis of the complete Valencian Sentinel Network data set**. Weekly influenza incidence rates (per 100.000 inhabitants) of all the seasons indicating in which weeks it is not possible to obtain the posterior probability (showing the weekly rate in black), in which ones it has not been obtained (in white) and, for those in which it has been calculated, if probability is greater than 0.5 (in red) or less than 0.5 (in blue).

Finally, if we kept obtaining the posterior probability of being in the epidemic phase for all the possible weeks in our data set, the second graph that FluDetWeb returns would have the appearance of Figure 7, in which all the weekly rates of all the seasons are colored as mentioned above, that is, black spots for those weeks in which it is not possible to obtain the posterior probability, white for those in which it has not been obtained, red for those with probability greater than 0.5 and blue for those lower than 0.5. As can be seen, our method provides very good results: it detects that the system is in an epidemic phase nearly always and it usually does it very close to the start of the epidemic.

## Conclusion

Our interest in this paper has been to describe an implementation of a prospective methodology for obtaining the posterior probability of being in an epidemic phase. Implementation has been done using a client-server architecture with a web-based client application design, which allows users to introduce and edit their own data, and obtain information about the possibility of their system being in an epidemic phase. Data needed are weekly ILI incidence rates (per 100000 habitants) provided by a Sentinel Network obtained by considering only the population covered by those sentinels that report information on the corresponding week. In order to obtain results, the minimum input dataset must contain at least 3 years of historical rates. Availability and software requirements are listed below in the following Section.

We now comment on possible extensions to this implementation. First of all, one of the benefits of using a three tier architecture in which the functions of the client-server are defined separately is that each layer could be upgraded or replaced independently. This modularity allows us to change any part we want, for instance, the algorithm used to detect the instant. We could change it, for example, for another in which the probability of being in an epidemic phase could depend not only on the rate of the previous week but also on the particular moment in the season (maybe at its early stages or at its final ones).

In line with this, at the moment we are developing a different methodology which could be used with other kinds of data (percentages, rates, etc.), for instance, with data coming from Sentinel Networks in which providers report weekly the percentage of patients with ILI from the total number of patients seen and the number of those patients with ILI.

Another extension could be to incorporate other statistical algorithms for automated monitoring of influenza surveillance and the possibility of comparing their behaviour, in a similar way as in the R-package surveillance by Höhle [28], which contains functionality to visualize surveillance data, provides algorithms for the detection of aberrations and benchmark numbers like sensitivity, specificity and detection delay in order to compare algorithms.

With respect to the limitations of this implementation, we should point out that our prospective influenza surveillance methodology needs the specification of two hyperparameters, a and b. Our web system has been fine-tuned for these values by giving two specific values. Using them in other situations could result in erroneous conclusions. The second limitation is the need of a complete run of the MCMC method every week. The waiting time for getting the result is not too long (less than 5 minutes), but a great demand of this system could cause a long delay in getting back the results. One way of solving this issue could be using sequential MCMC. This method basically consists of taking advantage of the results from the previous week in order to get more rapid an estimation of the probability of being in an epidemic phase in the analyzed week.

Finally, we would like to stress that the ease of use of FluDetWeb and its on-line availability can make it a valuable tool for public health practitioners who want to obtain information about the probability that their system is in an epidemic phase and that the architecture described can also be useful for developers of systems based on computationally intensive methods.

## Availability and requirements

Project name: FluDetWeb.

Project home page: http://www.geeitema.org/meviepi/fludetweb/

Operating system: Platform independent.

Programming language: R, WinBUGS, JavaServer Pages, Java (tested with Mozilla and Internet Explorer).

Other requirements: Java 1.3.1 or higher, Tomcat 4.0 or higher, Rserve, Java Mail, Rclient, JasperReport and MySQL.

License: GNU, GPL.

Any restrictions to use by non-academics: no licence needed.

## Competing interests

The authors declare that they have no competing interests.

## Authors' contributions

DC helped to conceive the application, participated in designing the application and drafted the manuscript. ALQ participated in designing the application, and helped with the program and drafting the manuscript. MAMB participated in designing the application, and helped with the program and with drafting the manuscript. MTM was responsible of acquisition of data and helped with drafting the manuscript. FV designed and programmed the application. All authors have read and approved the final manuscript.

## Pre-publication history

The pre-publication history for this paper can be accessed here:

http://www.biomedcentral.com/1472-6947/9/36/prepub

## Supplementary Material

Additional file 1 The zip file contains the data set (*sent-val-2008.dat*), a file with the WinBUGS model (*model.txt*), the ℝ codes (*functions.r *and *use-of-functions.r*) used in the web site and an instruction file (*instructions_R.doc*). With the provided information users can reproduce all the Figures in this document. Users can directly run the script with the ℝ software, following the directions in the instruction file, to obtain the graphical outputs. Click here for file
