# Supplementary material for: FluDetWeb: an interactive web-based system for the early detection of the onset of influenza epidemics
Source: BMC Med Inform Decis Mak. 2009 Jul 29;9:36. doi: 10.1186/1472-6947-9-36 (PMC2732617; doi:10.1186/1472-6947-9-36)
Supplement: Additional file 1 — The zip file contains the data set (sent-val-2008.dat), a file with the WinBUGS model (model.txt), the ℝ codes (functions.r and use-of-functions.r) used in the web site and an instruction file (instructions_R.doc). With the provided information users can reproduce all the Figures in this document. Users can directly run the script with the ℝ software, following the directions in the instruction file, to obtain the graphical outputs. [file 1472-6947-9-36-S1.zip › instructions_R.doc]

**Instructions for running the R scripts**

Here there are the instructions for running the R scripts:

- Create a work directory.
- Install R and WinBUGS.
- Put the 2 scripts (“*functions.r*” and “*use-of-funtions.r*”) there jointly with the file “*model.txt*” and the “*database sent-val-2008.dat*”.
- Run the script “*use-of-functions.r*” from R software.
